# Complex-shaped three-dimensional multi-compartmental microparticles generated by diffusional and Marangoni microflows in centrifugally discharged droplets

**DOI:** 10.1038/srep20793

**Published:** 2016-02-10

**Authors:** Masayuki Hayakawa, Hiroaki Onoe, Ken H. Nagai, Masahiro Takinoue

**Affiliations:** 1 Department of Computational Intelligence and Systems Science, Tokyo Institute of Technology, Yokohama, Kanagawa, 226-8502, Japan; 2 Department of Mechanical Engineering, Keio University, Yokohama, Kanagawa, 223-8522, Japan; 3 School of Materials Science, Japan Advanced Institute of Science and Technology, Nomi, Ishikawa, 923-1292, Japan; 4 PRESTO, Japan Science and Technology Agency, Kawaguchi, Saitama, 332-0012, Japan

## Abstract

We report a versatile method for the generation of complex-shaped three-dimensional multi-compartmental (3D-MC) microparticles. Complex-shaped microparticles have recently received much attention for potential application in self-assemblies, micromachines, and biomedical and environmental engineering. Here, we have developed a method based on 3D nonequilibrium-induced microflows (Marangoni and diffusional flows) of microdroplets that are discharged from the tip of a thin capillary in a simple centrifugal microfluidic device. The microparticle shapes can be tuned by the partial dissolution of specific compartments and by the deformation of the precursor microdroplets by manipulating the 3D microflows. We believe that this method will have wide applications in nano- and microscience and technologies.

Recently, microparticles have attracted much attention for use in a broad range of applications, including self-assembled materials^1^,^2^,^3^, self-propelled micromotors^4^,^5^,^6^,^7^, biomedical engineering of drug delivery systems^8^,^9^, and environmental engineering^10^. Complex-shaped microparticles with geometries that are non-spherical^11^,^12^,^13^, high-aspect-ratio^14^,^15^,^16^, or asymmetric^17^,^18^,^19^ have been developed^20^,^22^, which has further extended the range of applications of microparticles. Some research groups have reported microparticle fabrication methods based on the solidification of microdroplets deformed by controlling the contact angle of fluids in microfluidic devices^23^,^24^. An on-demand synthesis based on digital-microfluidic liquid handling was also reported^25^. These methods are useful, but the shapes and dimensions of the generated microparticles are limited (e.g., 2.5-dimensional ones). This limitation is attributed to the difficulty of controlling three-dimensional (3D) microflows during the process of microparticle generation. Therefore, it is necessary to develop a generation method that enables the construction of various-shaped three-dimensional multi-compartmental (3D-MC) microparticles by the effective control of 3D microflows.

In this study, we developed a versatile method for generating complex-shaped 3D-MC microparticles based on the effective control of 3D microflows and the partial dissolution of the microparticles (Fig. 1a). The synthesis was performed using a 3D capillary microfluidic device^26^ that can produce spherical multi-compartmental microparticles (Fig. 1b; details are given in Supplementary Fig. S1). The device was composed of a glass capillary, capillary holder, and disposable microtube; a CaCl_2_ solution was placed at the bottom of the microtube. When the device was centrifuged, the solution in the glass capillary dripped into and gelated in the CaCl_2_ solution at the bottom of the microtube (Fig. 1c). The produced microparticles had multiple compartments, depending on the barrel configuration of the glass capillary (Fig. 1d). This method can tune the shapes of the microdroplets using microflows induced by the nonequilibrium condition of the solutions before solidification (Fig. 1a), though no deformation is induced without microflows (Fig. 1a). For the deformation of droplets, we used a diffusional flow (Fig. 1a) and a Marangoni flow (Fig. 1a). The diffusional flow is driven by the difference in molecular concentration between the two solutions; the solution flows from higher- to lower-concentration regions. The Marangoni flow is driven by the difference in surface tension between the two solutions, and it flows from solutions with lower to higher surface tensions. Both microflows continued until the difference between the two solutions relaxed into the equilibrium state; thus, the degree of deformation could be kinetically tuned by controlling the time necessary for droplet solidification. After the solidification of the deformed microdroplets, some parts of them were dissolved, and finally complex-shaped 3D-MC microparticles were obtained. The diffusional flow was induced within the solution, while the Marangoni flow was induced on the surface of the solution. Thus, using the two types of flow, both the interior and the surface of the droplet can be deformed, hence enabling the construction of microparticles with complex shapes. The kinetic tunability by 3D deformation is one of the unique features of this method; previous methods generally produced equilibrated shapes after the completion of deformation.

## Results

### Synthesis of complex-shaped microparticles without microflows

**Figure 1:**
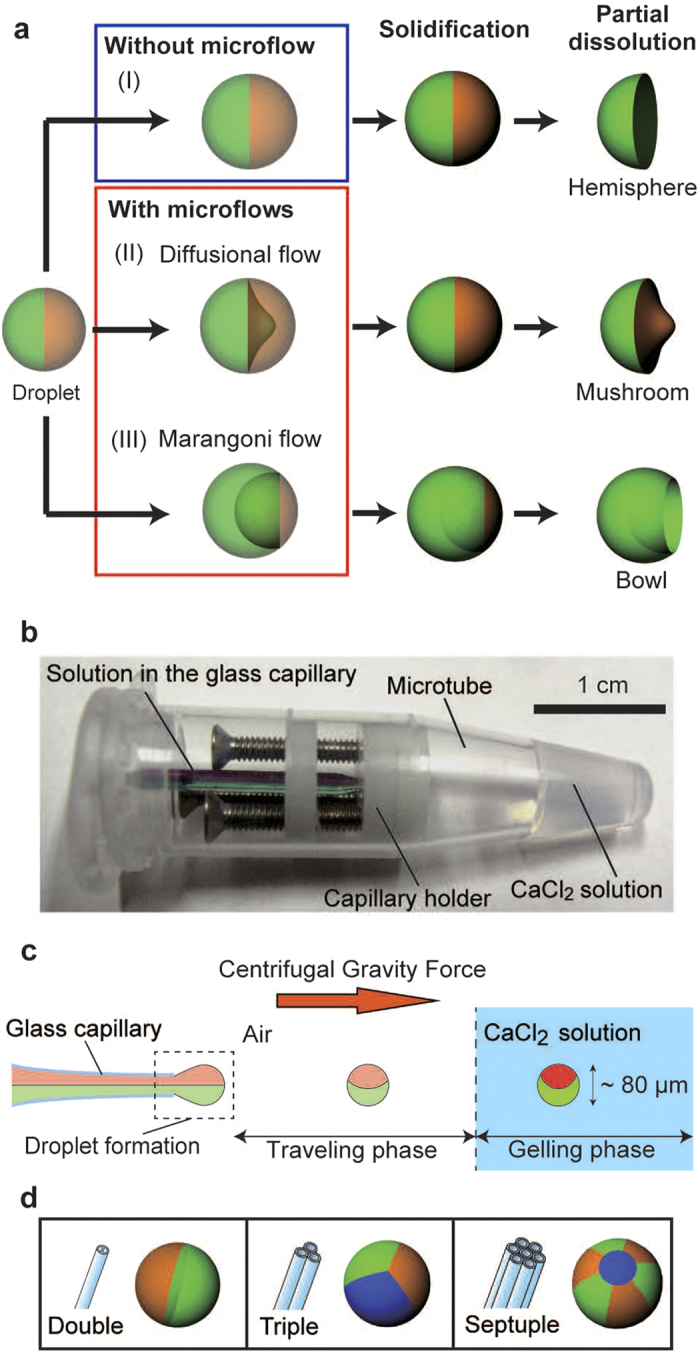
Conceptual illustrations of the synthesis of complex-shaped 3D-MC microparticles. (**a**) Complex-shaped microparticles obtained by partial dissolution of the spherical microparticles (I). More complex-shaped microparticles can be produced based on droplet deformation caused by nonequilibrium-induced solution microflows. When each compartment has a different composition concentration, diffusional flow occurs between compartments (II); when each compartment has a different surface tension, a Marangoni flow occurs (III); as a result, the microdroplets are deformed. After the partial dissolution of the deformed spherical microparticle, complex-shaped microparticles with geometries resembling mushrooms and bowls are obtained. (**b**) Picture of a 3D capillary microfluidic device. (**c**) Generation of spherical microparticles. Under centrifugal force, microdroplets are generated at the tip of the capillary and are shot into a CaCl_2_ solution (travelling phase). Then, the Na-alg solution in the microdroplets is solidified into Ca-alg gels in the CaCl_2_ solution (gelling phase). (**d**) Configurations of multi-barrelled capillaries and schematics of generated compartmentalized microparticles.

**Figure 2:**
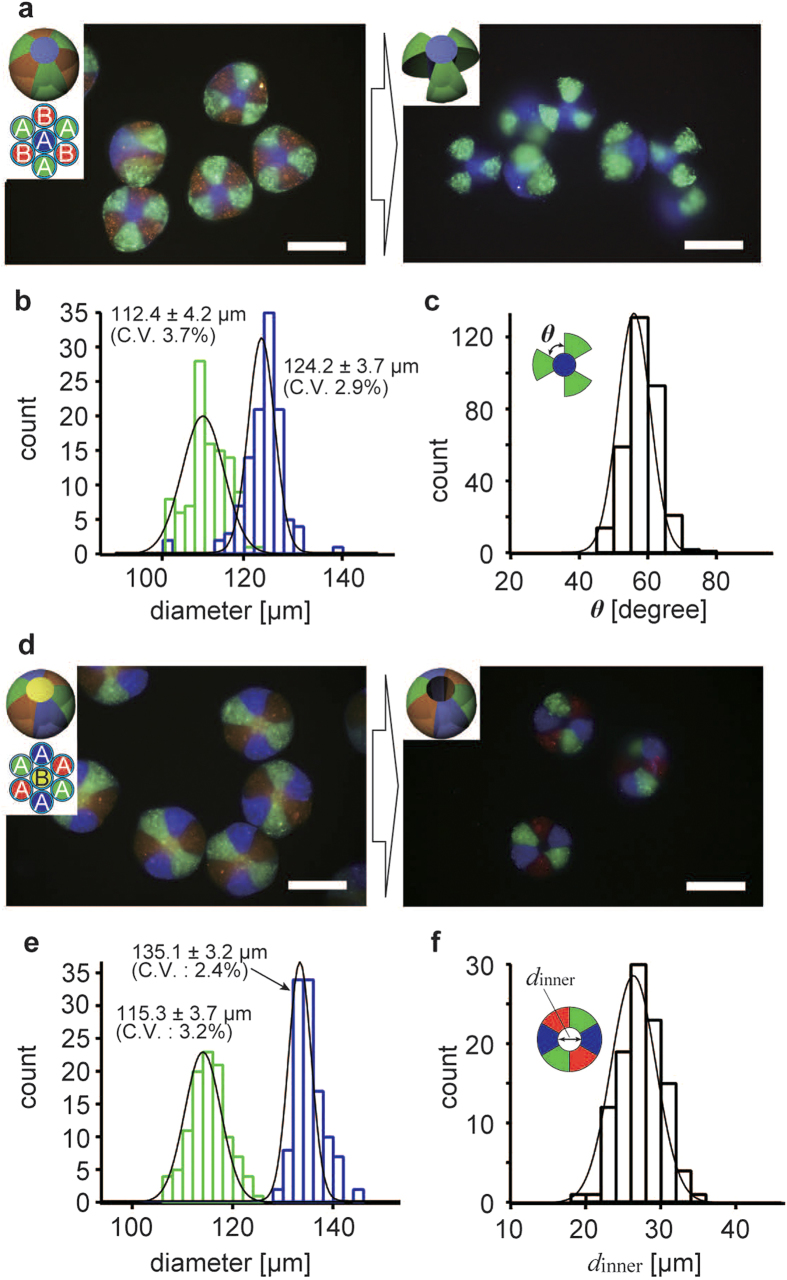
Observation of complex-shaped 3D-MC microparticles produced without microflows. (**a**) Fluorescence microscopy images of spherical 3D-MC microparticles (left) and triple-bladed propellers produced from them (right). Scale bars: 100 μm. Insets are illustrations of designs and configurations of multi-barrelled capillaries. Solutions A and B were coloured with 100 nm fluorescent nanobeads. The CaCl_2_ concentration for gelation was 3 M. (**b**) Histograms of the diameters of the spherical and the triple-bladed propeller-shaped microparticles. (**c**) Histograms of the inner angles *θ* of the triple-bladed propellers. (**d**) The production of doughnut rings. Scale bars: 100 μm. (**e**) Histograms of the diameters of the spherical and doughnut-ring particles. (**f**) Histogram of internal diameters of the doughnut rings.

We investigated the synthesis of 3D-MC microparticles by the partial dissolution of microparticles produced without microflows (Fig. 1a). Figure 2a shows fluorescence microscopy images of the produced triple-bladed propellers (right) and spherical 3D-MC precursor microparticles before the partial dissolution (left). The insets depict the microparticle designs and the configuration of the glass capillaries with the introduced solutions. Solution-A, composed of sodium alginate (Na-alg) and agarose solution, was introduced into the green and blue parts of a septuple glass capillary, and Solution-B (Na-alg solution) was introduced into the red parts. The solutions were coloured using fluorescent nanobeads. The agarose in Solution-A was intended to form the body of the complex-shaped microparticles, while Na-alg was used as a temporary gelating agent to fix the agarose into the appropriate compartment. When a centrifugal force (centrifugal acceleration *a* ~ 1000 × *g*) was applied to the capillary, the solutions dripped from the tip of the glass capillary (Fig. 1c). The microdroplets travelled through an air gap (travelling phase) and entered the CaCl_2_ solution. Then the Na-alg in each solution formed a calcium alginate (Ca-alg) gel and spherical 3D-MC microparticles were generated (gelling phase). To solidify the agarose, the microparticles were cooled. As a result, the green and blue parts formed an interpenetrating network gel of Ca-alg and agarose (Fig. 2a, left). After gelation, the Ca-alg gel in all parts was dissolved by removing the Ca^2+^ ions with the Ca-chelating agent of ethylenediamine-tetraacetic acid (EDTA). After chelation, we obtained triple-bladed propellers containing solely agarose gel (Fig. 2a, right). Figure 2b presents the size distributions of the spherical precursor microparticles (blue) and the partially dissolved microparticles (green). The coefficient of variation (C.V.) was ~3%. The diameters of the partially dissolved microparticles were smaller than those of the spherical precursor microparticles. Based on the Flory theory of the swelling equilibrium of gels^27^, the volume of the gel is determined by the competition between the elastic force and the osmotic pressure on the gels. In this case, it is considered that the elution of the Na-alg gel induced changes in the elastic force and the osmotic pressure acting on the hydrogel microparticle, resulting in the volume change of the microparticles. The angle *θ* of the propeller blades was also measured with a C.V. of ~10% (Fig. 2c). This low C.V. suggests that the shape of the complex-shaped microparticles was relatively monodisperse, as well as the diameters of the particles. A variety of complex shapes, including doughnut rings (Fig. 2d), two-thirds of spheres, and double-bladed propellers (see Supplementary Fig. S2), were also successfully synthesized as 3D-MC microparticles. These particles also had small C.V.s, similar to that of the triple-bladed propellers (Fig. 2e,f, and Supplementary Fig. S2).

### Deformation of spherical microparticles by nonequilibrium-induced microflows

**Figure 3:**
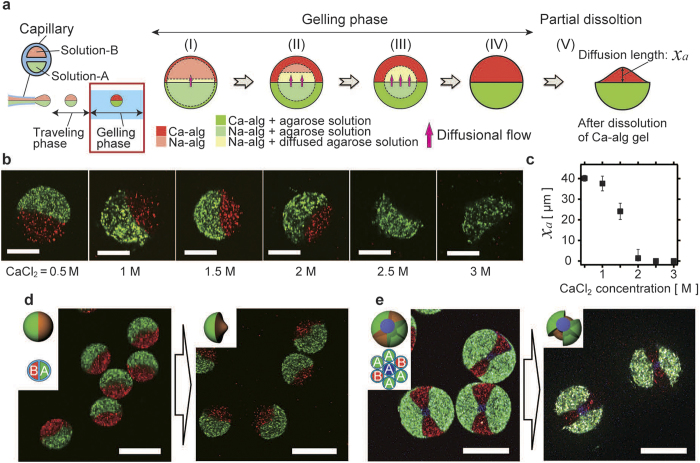
Investigation of deformation induced by diffusional flow and extension to the synthesis of 3D-MC complex-shaped microparticles. (**a**) The deformation mechanism from diffusional flow. When there is a difference in agarose concentrations between each compartment of a microdroplet, a diffusional flow of agarose is generated during the travelling and gelling phase. The front of the sodium alginate gelation develops from the surface of the microdroplets, and the diffusional flow is interrupted. The diffusional flow of agarose produces deformation inside the microdroplets. Finally, deformed microparticles are obtained. (**b**) CLSM images of agarose microparticles deformed by diffusional flow. Scale bars: 50 μm. (**c**) Dependence of *x*_*a*_ on CaCl_2_ concentrations. The error bars denote the standard deviations. (**d,e**) CLSM images of spherical 3D-MC microparticles deformed by diffusional flow (left) and complex-shaped 3D-MC microparticles produced from them (right). (**d**) Mushroom-shaped microparticles. (**e**) Propeller-shaped microparticles with thick axes. Scale bars: 100 μm.

We synthesized complex-shaped 3D-MC microparticles by deforming the microdroplets using a nonequilibrium-induced diffusional microflow (Fig. 1a). The deformation scheme is summarized in Fig. 3a. The green part included Solution-A and the red part included Solution-B; because of the difference in the concentration of agarose between the two parts, the agarose molecules diffused from the green part to the red part (Fig. 3a and Supplementary Fig. S3). This process was expected to occur in the gelling phase, because the duration of the traveling phase was short. The time for traveling (~10^−4^ s) was predicted by the velocity of the shot droplet obtained from the terminal velocity (details given in Supplementary Fig. S1). This travelling phase was short compared to the diffusion time (~10^1^ s) predicted using the diffusion constant of the agarose molecule. During the gelling phase, Na-alg solutions in both parts were solidified from the surface of the droplets (Fig. 3a) because of the diffusional permeation of Ca^2+^ ions into the droplet. The diffusion of agarose molecules into the gelled area was very slow compared to that in the non-gelled area; hence, it almost stopped at the gelating front (Fig. 3a). Finally, when all the Na-alg in the droplet had formed Ca-alg (Fig. 3a), the locations of the diffused agarose molecules were fixed. After the gelation of agarose by cooling and the dissolution of the Ca-alg gel, diffusionally expanded agarose gel particles were obtained (Fig. 3a (V)). Figure 3b shows confocal laser scanning microscopy (CLSM) images of the agarose microparticles. The red-fluorescent region was generated by the diffusional spreading of agarose, which only occurred when the CaCl_2_ concentration was low (Fig. 3b, 0.5–2 M). When the CaCl_2_ concentration exceeded 2 M, diffusional spreading of agarose was not observed. Figure 3c shows the dependence of the diffusional spreading length *x*_*a*_ (defined by Fig. 3a (V)) of agarose on the CaCl_2_ concentration. *x*_*a*_ is observed to decrease as the CaCl_2_ concentration increases. As a result, the deformation was successfully controlled by manipulating the CaCl_2_ concentration. Figure 3d,e show CLSM images of the spherical 3D-MC microparticles produced with the diffusional flow of agarose at 1.5 M CaCl_2_ (left of each panel) and complex-shaped microparticles produced by the partial dissolution of the spherical particles (right of each panel): a mushroom-like shape (Fig. 3d) and a double-bladed propeller shape with a thick axis (Fig. 3e).

**Figure 4:**
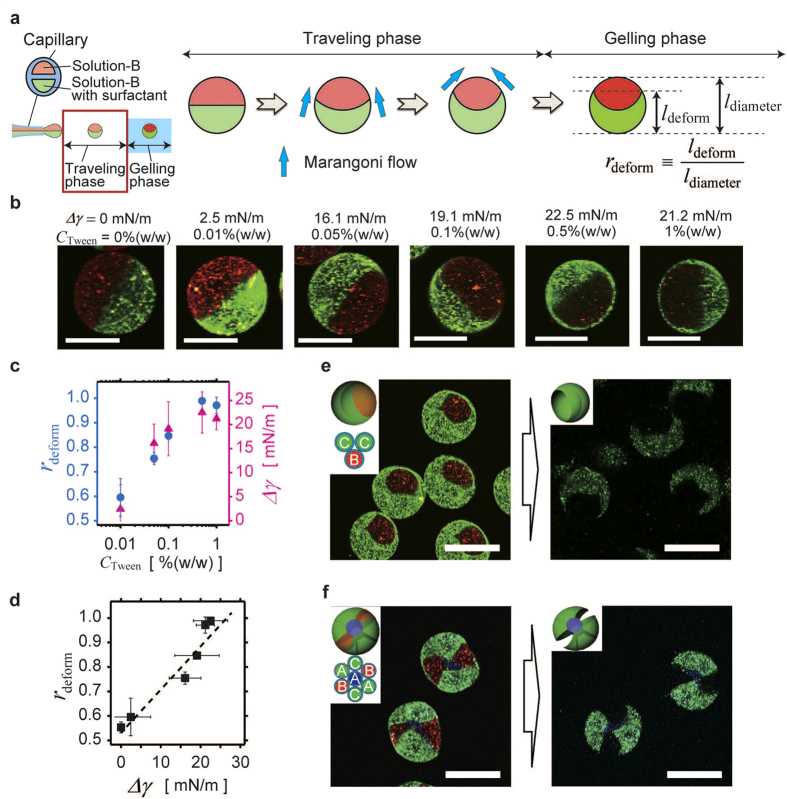
Investigation of deformation induced by Marangoni flow and extension to the synthesis of 3D-MC complex-shaped microparticles. (**a**) Deformation mechanism from Marangoni flow. When there is a difference in surface tension between each compartment of a microdroplet, Marangoni flow occurs during the travelling phase. The solution flows from a compartment having a lower surface tension to that with a higher surface tension. Marangoni flow deforms the surface of the microdroplets. (**b**) CLSM images of Ca-alg gel microparticles deformed by a Marangoni flow induced by surface tension differences Δ*γ*. Scale bars: 50 μm. (**c**) Dependence of *r*_deform_ on Tween 20 concentration (*C*_Tween_) (blue circle) and dependence of Δ*γ* on *C*_Tween_ (magenta triangle). (**d**) Relationship between Δ*γ* and *r*_deform_. In (**c**,**d**), the error bars denote the standard deviations. (**e,f**) CLSM images of spherical 3D-MC microparticles (left) and 3D-MC complex-shaped microparticles produced from them (right). (**e**) Bowl-shaped microparticles. (**f**) Propeller-shaped microparticles with sharp edges. Scale bars: 100 μm.

Next, we synthesized complex-shaped 3D-MC microparticles using the deformation of microdroplets induced by the nonequilibrium-induced Marangoni microflow (Fig. 1a). The deformation scheme is shown in Fig. 4a. The red part included Solution-B, and the green part included Solution-B with a surfactant (Tween 20) to decrease the surface tension of the green part compared to that of the red part. During the travelling phase, the surface of the droplet was deformed by the Marangoni flow. The required time for the Marangoni flow to attain equilibrium was estimated to be ~10^−4^ s (Supplementary Fig. S4), which was comparable to the duration of the traveling phase (~10^−4^ s). The red part (higher surface tension) was covered with the green part (lower surface tension) and the deformation was immediately stopped by surface gelation of the droplet in the gelling phase. Figure 4b shows CLSM images of Ca-alg two-faced (Janus) microparticles deformed by the Marangoni flow under various surface tension differences, Δ*γ*, as tuned by manipulating the concentration of Tween 20 (*C*_Tween_). The deformation ratio *r*_deform_ (as defined by Fig. 4a) increases with increasing *C*_Tween_ and reaches saturation at *C*_Tween_ ~0.5% (w/w) (Fig. 4c, blue circle). The saturation point was comparable to the saturation point of Δ*γ* (Fig. 4c, magenta triangle). Figure 4d shows that *r*_deform_ was proportional to Δ*γ*, suggesting that the deformation induced by Marangoni flow can be controlled by varying the difference in surface tension.

Here, the Marangoni-flow-induced deformation is theoretically investigated (see also Supplementary Fig. S4). The solutions are driven by the force induced by the surface tension difference *f* ~ Δ*γ*/*r*, where *r* is the radius of the sol droplet. In the steady state, *f* is balanced by the shear stress τ = −η *v*_*x*_/*r*, where η and *v*_*x*_ are the viscosity coefficient of the sol and the velocity of the Marangoni flow, respectively. Thus, we obtained the relation *v*_*x*_ = −Δ*γ*/η, hence *v*_*x*_ ∝ Δ*γ*. Because *r*_deform_ depends on the migration length of the front of the Marangoni flow, *r*_deform_ ∝ Δ*γ*. These theoretical results agree with the experimental results. Figure 4e, f show CLSM images of the spherical 3D-MC microparticles deformed by the Marangoni flow (left of each panel) and the complex-shaped microparticles produced from the spherical precursors (right of each panel). Solution-A, Solution-B, and Solution-C (a solution of Solution-A with Tween 20) were used, and only the green parts of Solution-C were deformed by the Marangoni flow. Here, the complex-shaped microparticles having bowl-like shapes (Fig. 4e) and double-bladed propeller shapes with sharp edges (Fig. 4f) were synthesized.

**Figure 5:**
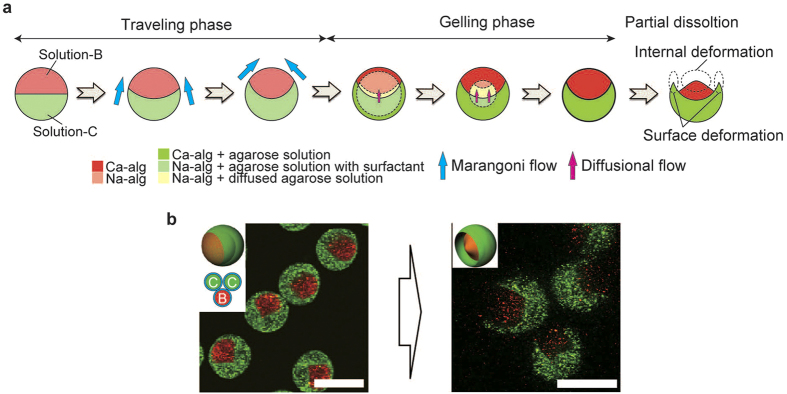
The synthesis of 3D-MC complex-shaped microparticles based on the combination of diffusional flow and Marangoni flow. (**a**) The deformation mechanism of the combination of diffusional flow and Marangoni flow. The microdroplet is firstly deformed by the Marangoni flow during the travelling phase. While the deformation of the Marangoni flow stops in the gelling phase, the diffusion of agarose molecule continues and deforms the internal structures of the microdroplet until the flow of fluid is interrupted by the formation of Ca-alg gel. Consequently, complex-shaped microparticles with both internal and surface deformations are obtained. (**b**) CLSM images of spherical 3D-MC microparticles deformed by diffusional flow and Marangoni flow (left) and complex-shaped 3D-MC microparticles produced from them (right). Scale bars: 100 μm.

Finally, we synthesized complex-shaped 3D-MC microparticles using both diffusional and Marangoni flows. The scheme of deformation is summarized in Fig. 5a. Here, the red part contains Solution-B, and the green part contains Solution-C. The concentration of the CaCl_2_ solution for solidification was 1.5 M. First, Solution-B was covered with Solution-C; the surface of the droplet was deformed because of the surface tension difference during the travelling phase. Then, in the gelling phase, the agarose molecules in Solution-C diffused into Solution-B while the Na-alg in both solutions were gelled from surface of the droplet. After the cooling of the agarose solution, spherical 3D-MC microparticles were obtained (CLSM image in Fig. 5b, left). Finally, the Ca-alg gel in all parts was dissolved by the addition of EDTA solution; complex-shaped 3D-MC microparticles with deformed interior and surface structures were obtained (Fig. 5b, right).

## Discussion

In this work, we developed a versatile method for generating complex-shaped 3D-MC microparticles. We successfully controlled the shapes of the microparticles using deformation from induced diffusional and Marangoni flows. During both deformation mechanisms, we captured instantaneous views of the nonequilibrium states of the microdroplets influenced by microflows at given times. We showed that the dominant control parameter for deformation using the diffusional flow was the gelling speed of the Na-Alg, which was controlled by varying the Ca^2+^ concentration (Fig. 3b,c and Supplementary Fig. S3); we also showed that the dominant control parameter for deformation using the Marangoni flow was the surface tension difference between the solutions, which was controlled by changing the concentration of the surfactant (Fig. 4d and Supplementary Fig. S4). These parameters directly controlled the microflow kinetics in the precursor microdroplets. The kinetically tuneable deformation in the respective travelling and gelling phases is greatly advantageous in this generation method. Because the microflow-induced deformations occur in different phases of the synthesis reaction, microparticles with both internal and surface deformations were successfully produced by the application of both induced flows. We found that the contours of the structures deformed by the diffusional flow were slightly blurred in comparison with the other structures (Figs 3b,d,e and 5b). We consider that the density of the agarose gel, originating from the front of the diffusional flow, was somewhat lower than that present in the other parts, and was insufficient to maintain the embedded fluorescent nanobeads. In addition, the thin agarose films produced at the front of the flow were slightly damaged in the process of dissolving the Ca-alg gel, as shown in Figs 4e and 5b. We anticipate that these structural issues with the agarose gel may be solved by the use of other gels with higher cross-linking densities and degrees of robustness. Our method can be generally applied to various materials such as N-isopropylacrylamide (NIPAAm), polyethylene(glycol)diacrylate (PEGDA), and collagens, but the following conditions are required to control the microparticle shapes accurately: (i) The gelling mechanisms of the two solutions should be different; (ii) The time scale of gelation should be comparable to that of diffusional flow to control the deformation by the diffusional flow; (iii) The time scale of the traveling phase should be comparable to that of the Marangoni flow to control the deformation by the Marangoni flow.

The method described here may be significant for the development of microparticle-based applications such as self-assembled materials^1^,^2^,^3^, self-propelled micromotors^4^,^5^,^6^,^7^, drug delivery systems^8^,^9^, and environmental engineering^10^. As an example, we have demonstrated the application of this method in designing self-propelled micromotors exhibiting controlled directional motions due to their asymmetric shapes (Supplementary Fig. S5). For further development, this method will contribute to the fabrication of various integrated systems of microparticles using functional materials such as pH-responsive DNA^28^,^29^, molecular memories^30^, and computers^31^. Such integrated functional complex-shaped microparticles will realize higher-order self-assembly based on specific interactions and computations by programmed DNA sequences, highly functional drug delivery systems, and the development of autonomous molecular robots^32^,^33^ with sensing and information-processing functions. We believe that this synthesis method will promote the use of microparticle-based applications in several fields of science and technology.

## Methods

### Fabrication of centrifugal capillary-based microfluidic device

The capillary-based microfluidic device was composed of a glass capillary, a microtube, and capillary holders made of polyacetal resin (Supplementary Fig. S6). Both the glass capillaries (World Precision Instruments, TST150-6, 3B120F-4, and 7B100F-6) and the microtube (BIO-BIK, 1.5 mL microtube CF-0150) were obtained commercially. The tip of the glass capillary was sharpened by a puller (NARISHIGE, PC-10) and the capillary orifice was tuned by a Microforge (NARISHIGE, MF-900). Using a MODERA MDX-40 (Roland DG), 2-mm-thick polyacetal resin plates were cut into circular plates, and holes for the glass capillary and screw holes were drilled. The size of the central fixing hole varied depending on the thickness of the glass capillaries being used. The processed plates were assembled with screws (M2 × 1.5).

### Compositions of Solution-A, -B, and -C

Solution-A: a mixture of 2% (w/w) sodium alginate (Wako Pure Chemical Industries) and 1.5% (w/w) agarose (Sigma-Aldrich, Type IX-A, Ultra-low Gelling Temperature). Solution-B: 3% (w/w) sodium alginate solution. Solution-C: a mixture of 2% (w/w) sodium alginate, 1.5% (w/w) agarose, and 0.1% (w/w) polyoxyethylene (20) sorbitan monolaurate (Tween 20) (Wako Pure Chemical Industries).

### Fluorescent colouring of Solutions A–C

The 3D-MC microparticles shown in Figs 2, 3, 4 were coloured by fluorescent nanobeads; the compositions of each colour are as follows. Red: 0.1% (w/w) red-fluorescent nanobeads (Polysciences, 590 nm, #18719). Green: 0.1% (w/w) green-fluorescent nanobeads (Polysciences, 540 nm, #16662). Blue: 0.1% (w/w) blue-fluorescent nanobeads (Polysciences, 468 nm, #19774). Yellow: 0.05% (w/w) green-fluorescent nanobeads and 0.05% (w/w) red-fluorescent nanobeads.

### Synthesis and observation of complex-shaped microparticles

Sodium alginate and agarose were dissolved in pure water (Milli-Q). To avoid the premature gelation of agarose, both solutions were mixed after being heated to 80 °C. These solutions were reheated to 80 °C before introduction into the glass capillary. Microparticles obtained using the capillary-based microfluidic device were immediately cooled to 4 °C, and the agarose was solidified at 4 °C for 20 min. Subsequently, the microparticles were carefully washed with and soaked in pure water for approximately 1 h. Next, calcium alginate gels in the microparticles were dissolved by the addition of the EDTA solution with a final concentration of 0.25 M (Wako Pure Chemical Industries). These microparticles were observed using a fluorescence microscope (OLYMPUS, IX81) and a confocal laser scanning microscope (CLSM) (OLYMPUS, FV1000). In all experiments, a swing rotor centrifuge (HITECH, ATT 101) was used, and the centrifugal duration was 3 min at ~4200 rpm (~1000×*g*).

### Surface tension measurement

The surface tension of the sodium alginate solution containing Tween 20 was measured using the pendant-drop method^34^. We calculated the surface tension by analysing a photograph of the shape of a drop of the solution hanging from a pipette tip (WATSON, 122–703C) taken by a digital camera (Ricoh Company, R8).

## Additional Information

**How to cite this article**: Hayakawa, M. *et al*. Complex-shaped three-dimensional multi-compartmental microparticles generated by diffusional and Marangoni microflows in centrifugally discharged droplets. *Sci. Rep.* **6**, 20793; doi: 10.1038/srep20793 (2016).

## Supplementary Material

Supplementary Information

Supplementary Video S1

Supplementary Video S2
