# Supplementary material for: Complex-shaped three-dimensional multi-compartmental microparticles generated by diffusional and Marangoni microflows in centrifugally discharged droplets
Source: Sci Rep. 2016 Feb 10;6:20793. doi: 10.1038/srep20793 (PMC4748296; doi:10.1038/srep20793)
Supplement: Supplementary Information [file srep20793-s1.pdf]

Supplementary Information

Complex-shaped three-dimensional multi-compartmental microparticles generated by diffusional and Marangoni microflows in centrifugally discharged droplets

Masayuki Hayakawa[1], Hiroaki Onoe[2], Ken H. Nagai[3], and Masahiro Takinoue[1,4,*]

[1]Department of Computational Intelligence and Systems Science Tokyo Institute of Technology, Yokohama, Kanagawa, 226-8502, Japan.

[2]Department of Mechanical Engineering, Keio University, Yokohama, Kanagawa, 223-8522, Japan.

[3]School of Materials Science, Japan Advanced Institute of Science and Technology, Nomi, Ishikawa, 923-1292, Japan.

[4]PRESTO, Japan Science and Technology Agency, Kawaguchi, Saitama, 332-0012, Japan.

*takinoue.m.aa@m.titech.ac.jp

Supplementary Figures

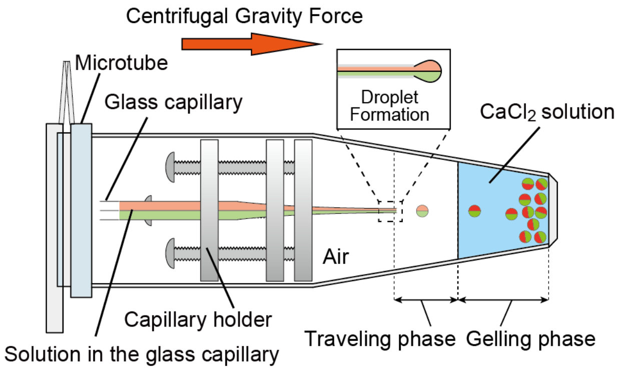

Figure S1. Illustration of the capillary-based microfluidic device we have reported previously[1].

The capillary-based microfluidic device is composed of a glass capillary, capillary holder, and disposable microtube. A $CaCl_2$ solution is put into the bottom of the microtube. Under a centrifugation, (centrifugal acceleration a : $1000\times g$) microdroplets are generated from the tip of the capillary and travel the air gap at terminal velocity u into a $CaCl_2$ solution at the bottom of the microtube (travelling phase). The motion of the sol droplet (Volume: V, Reference area: S) obeys the equation $\rho_s V \frac{du}{dt} = (\rho_s - \rho_f)Va - \frac{C_D \rho_f u^2 S}{2}$, where ρ_s, ρ_f are density of the sol droplet and the fluid respectively. C_D is the drag coefficient that varies depending on the Reynolds number Re, $C_D = \frac{24}{\mathrm{Re}}(1+0.27\,\mathrm{Re})^{0.43} + 0.47[1-\exp(-0.04\,\mathrm{Re}^{0.38})]$[2]. Here, we assume the Reynolds number is very low ($\mathrm{Re} = 1$), and in this case, C_D : $\frac{24}{\mathrm{Re}} = \frac{24\mu_f}{du_{\mathrm{Re}=1}\rho_f}$, where d is the diameter of the sol

droplet and μ_f is the viscosity coefficient of fluid. Since $\frac{du}{dt}=0$ at equilibrium, thus, we obtained so called Stokes' low $u_{\mathrm{Re}=1}=\frac{d^2\left(\rho_s-\rho_f\right)a}{18\mu_f}$. From this equation, the velocity of the sol droplet $u_{\mathrm{Re}=1}$ is estimated to be on the order of ~10^1 m/s. However, the Reynolds number obtained using $u_{\mathrm{Re}=1}$ is $\mathrm{Re}=\frac{du_{\mathrm{Re}=1}\rho_f}{\mu_f}$: 10^1, and means that this terminal velocity based on Stokes' low is not appropriate for our system. Therefore, discussion from the other range of the Reynolds number ($2<\mathrm{Re}<500$) is required. In this range, we use C_D : $\frac{24}{\mathrm{Re}}(1+0.27\,\mathrm{Re})^{0.43}$: $\frac{10}{\sqrt{\mathrm{Re}}}=10\sqrt{\frac{\mu_f}{du_{2<\mathrm{Re}<500}\,\rho_f}}$, hence, $u_{2<\mathrm{Re}<500}=\left\{\frac{4}{225}\frac{\left(\rho_s-\rho_f\right)^2a^2}{\mu_f\rho_f}\right\}^{\frac{1}{3}}d$ is derived. According to this formula, $u_{2<\mathrm{Re}<500}$ is ~10^1 m/s, and the Reynolds number using $u_{2<\mathrm{Re}<500}$ is in the range that we assumed ($2<\mathrm{Re}<500$). Thus, although the time scale of terminal velocities obtained from $u_{\mathrm{Re}=1}$ and $u_{2<\mathrm{Re}<500}$ are comparable, the velocity of the sol droplet should be considered using $u_{2<\mathrm{Re}<500}$. Due to the distance between the tip of glass capillary and the suface of the $CaCl_2$ solution is ca. 4 mm, we have the time scale of the traveling phase, ~10^{-4} s. Then, the Na-alg solution in the microdroplets was solidified into Ca-alg gels in the $CaCl_2$ solution (gelling phase).

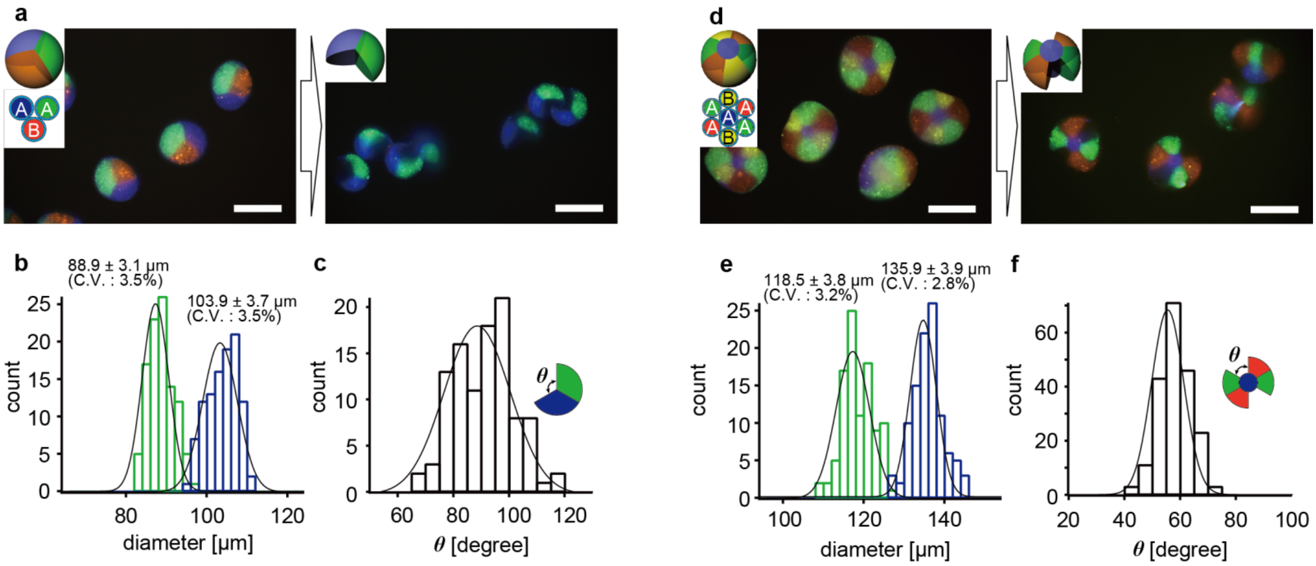

Figure S2. Generation of complex-shaped 3D-MC microparticles and their monodispersity.

(**a**, **d**) Fluorescence microscopy images of spherical 3D-MC microparticles (left) and complex-shaped 3D-MC microparticles produced from them (right). Insets are illustration of designs and configurations of multi-barreled capillaries. Solution-A, and -B were coloured with 100 nm fluorescent nanobeads. The $CaCl_2$ concentration for gelation was 3 M. (**b**, **e**) Size distributions of the precursor spherical microparticles (blue) and the partially removed microparticles (green). (**c**, **f**) Histograms of inner angles of the complex-shaped microparticles. (**a**-**c**) Two-thirds of spheres. (**d**-**f**) Double-bladed propellers.

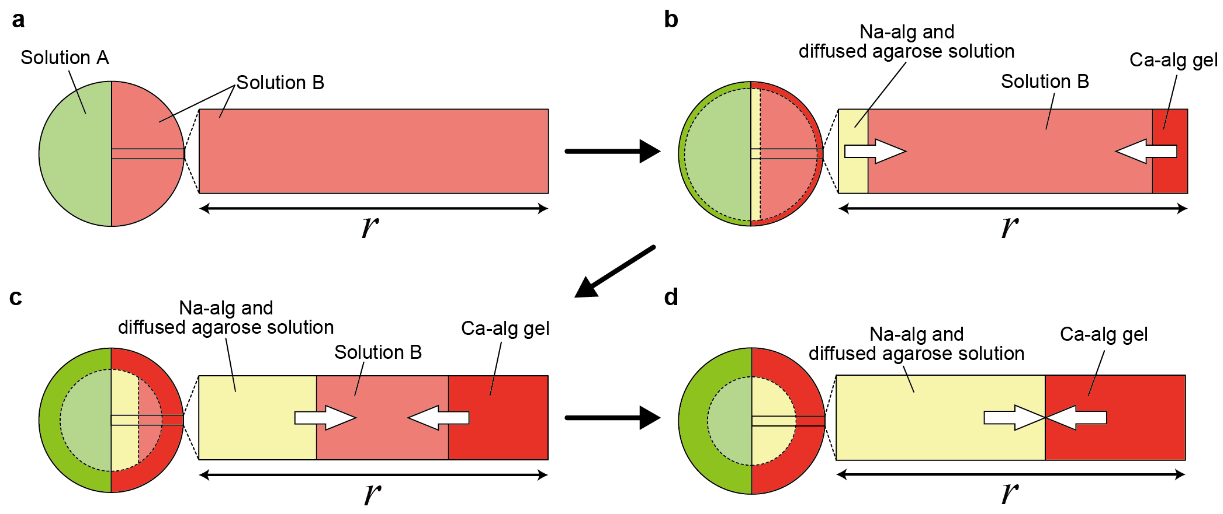

Figure S3. Generation of the diffusional flow inside the solution droplet.

Using the formula $\langle r^2 \rangle = 4Dt$ which is obtained from the diffusion equation, the time scale is estimated. Since the radius of particle is around 50 μm, it takes ~10^1 s for the front of the Ca-alg gel to reach the center of the droplet; the time is much longer than that of the traveling phase. From Solution-A (**a**), the agarose solution diffused into Solution-B (**b**,**c**). In the Ca-alg gel, diffusion of the agarose solution is finally interrupted (**d**).

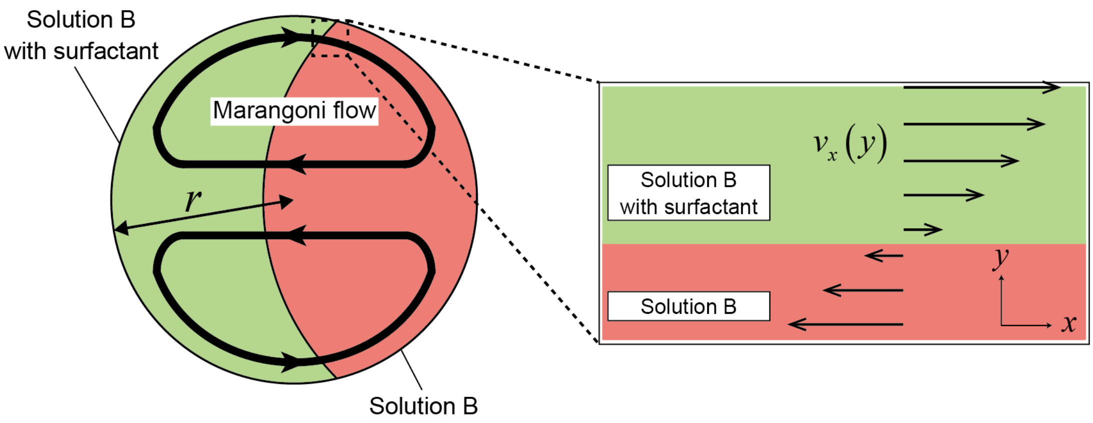

Figure S4. Generation of Marangoni flow on the surface of the solution droplet.

Here, the Marangoni-flow-induced deformation is investigated theoretically. The solutions are driven by surface tension difference $f = \frac{\partial \gamma}{\partial x}$. In a steady state, f is balanced by the shear stress $\tau = -\eta \frac{\partial v_x}{\partial y}$, where η is viscosity coefficient, thus, $0 = -\eta \frac{\partial v_x}{\partial y} + \frac{\partial \gamma}{\partial x}$. Since $f = \frac{\partial \gamma}{\partial x} : \frac{\Delta \gamma}{r}$ and $\tau = -\eta \frac{\partial v_x}{\partial y} : -\eta \frac{v_x}{r}$, where r is radius of the sol droplet, we obtained $v_x = -\frac{\Delta \gamma}{\eta}$. Hence $v_x \propto \Delta \gamma$. The time scale of the generation of the Marangoni flow also obatained using $v_x = -\frac{\Delta \gamma}{\eta}$. It is required that front of the Marangoni flow covers the surface of 75 μm (the quarter of the circumference of the microparticle with 100 μm diameter) to attain the equilibrium, thus the time scale of the Marangoni flow, ~ 10^{-4} s was derived using $\Delta \gamma$ showen in Figure 4b.

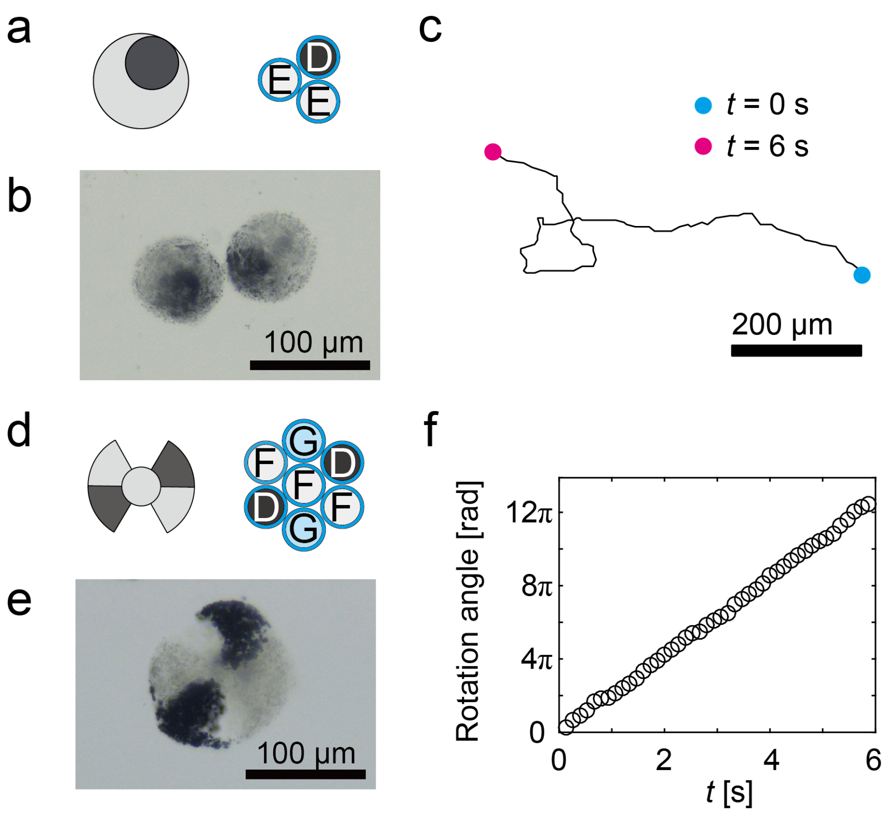

Figure S5. Translational, rotational, and circular motions of complex-shaped microparticles propelled by a catalytic reaction.

As an application of our synthetic method, self-propelled catalytic micromotors with various trajectories were synthesized. The synthesis and experimental conditions are given in the Supplementary Methods. (**a**) Schematic illustration of the deformed micromotors and capillary configurations. The dark gray area shows the catalytic cite containing platinum nanopowder (Pt). (**b**) Deformed spherical microparticles taken by a digital microscope. The agarose gel including Pt was covered with the agarose gel including polystyrene nanoparticles by the Marangoni-flow-induced deformation. (**c**) Trajectory of the microparticle of (**b**). The microparticle was propelled with bubbles resulting from the decomposition of hydrogen peroxide accelerated by the Pt catalyst, and thus

exhibited a directional translational motion because of the asymmetric localization of Pt (see also Supplementary Movie S1). Blue filled circle: the initial position (t = 0 s). Magenta filled circle: the position at t = 6 s. (**d**) Schematic illustration of the propeller-shaped micromotors and capillary configurations. (**e**) Double-propeller-shaped microparticle including Pt alternately in the propeller blades. The rotational symmetry of the microparticles was broken. (**f**) Time variation of the rotation angle of the microparticles with double Pt sites. The propellers induced a torque to the microparticle body, and thus unidirectional rotational motion occurred (see also Supplementary Movie S2).

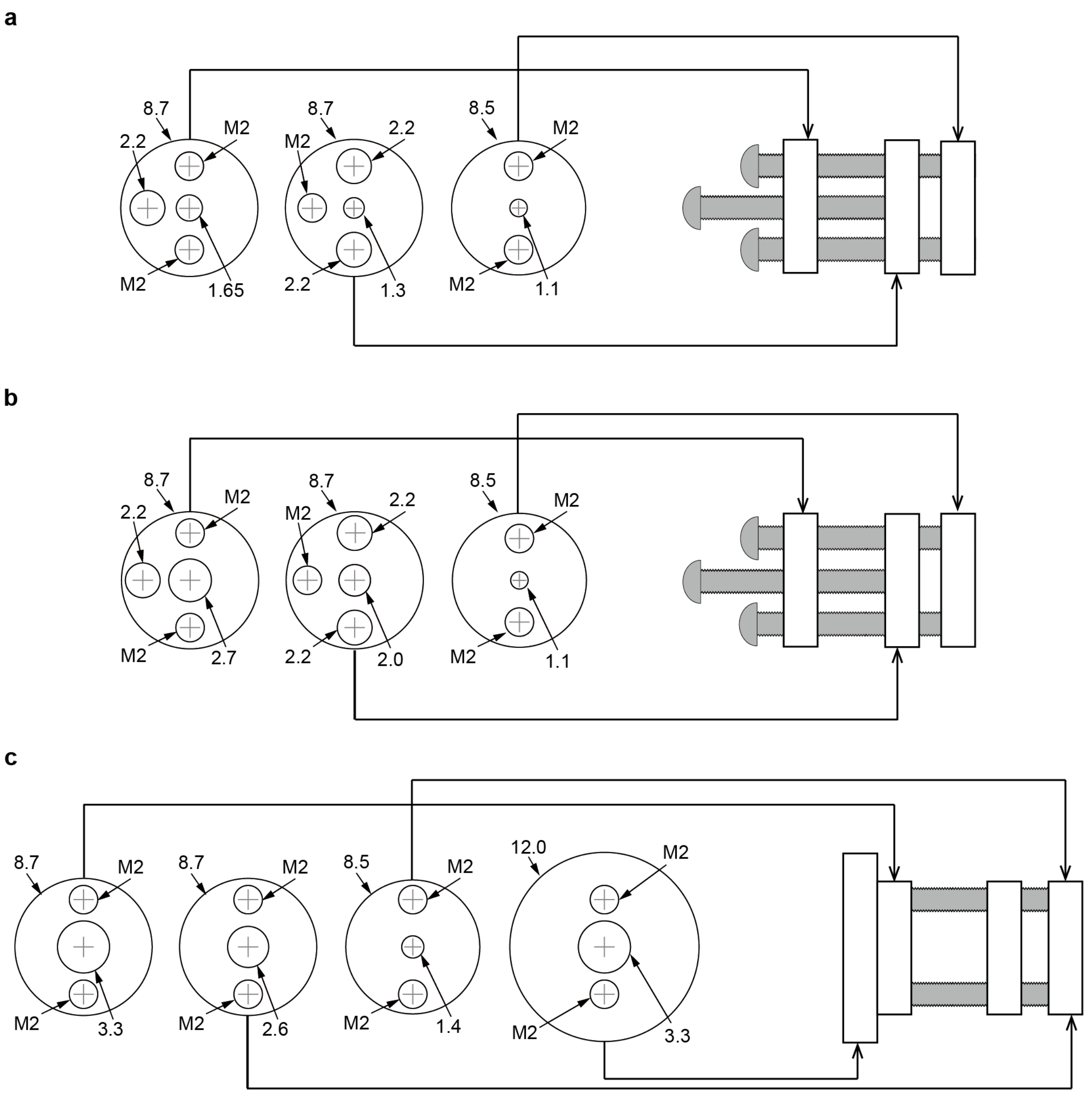

Figure S6. 2D schematic of the capillary holder and the assembled holder. The capillary holder for double-barreled (**a**), triple-barreled (**b**), and septuple-barreled (**c**) capilliaries. Each plate is made of polyacetal resin and is 2 mm thick.

Supplementary Movie

Movie S1. The movie data for Fig. S5c.

The deformed spherical microparticles having the Pt catalytic site showed directional transrational motion. The reproduction speed is real time speed.

Movie S2. The movie data for Fig. S5f.

The double-propeller-shaped microparticle with double Pt sites showed unidirectional rotational motion because of the rotational symmetry breaking. The reproduction speed is real times speed.

Supplementary Methods

Fabrication of self-propelled catalytic micromotors

To synthesize deformed spherical microparticles containing Pt, we used a mixture of Na-alg and agarose with Pt (Solution-D), and a mixture of Na-alg and agarose with polystyrene nanobeads (PSBs) (Solution-E), which has a lower surface tension. These solutions were introduced into a triple-barrelled glass capillary as shown in Supplementary Fig. S5. To synthesize propeller-shaped microparticles with double Pt sites, we introduced Solution-D, a mixture of Na-alg and agarose with PSBs (Solution-F), and a mixture of Na-alg and TritonX-100 (Solution-G) into the septuple-barrelled glass capillary as shown in Supplementary Fig. S5. Here, PSBs were added to facilitate optical observation under a digital microscope (KEYENCE, VHX-2000). To prevent aggregations, the platinum powder (Sigma-Aldrich, platinum powder, ≤10 μm) was dispersed into a 10% (w/w)

TritonX-100 solution under sonication for two hours before mixing into the Na-alg solutions. Detailed compositions of Solution-D, Solution-E, Solution-F, and Solution-G are shown below.

Compositions of Solutions-D,-E,-F, and-G

Solution-D: A mixture of 2% (w/w) sodium alginate, 1.5% (w/w) agarose, 10% (w/w) platinum powder (Sigma-Aldrich, platinum powder, ≤10 μm), and 0.1% (w/w) polyoxyethylene (10) octylphenyl ether (TritonX-100) (Wako Pure Chemical Industries). TritonX-100 was added to inhibit platinum powder aggregation. Solution-E: A mixture of 2% (w/w) sodium alginate, 1.5% (w/w) agarose, 0.5% (w/w) 1.0 μm Polystyrene microbeads (PSBs) (Polysciences), and 1% (w/w) TritonX-100. Solution-F: A mixture of 2% (w/w) sodium alginate, 1.5% (w/w) agarose, 0.5% (w/w) PSBs, and 0.1% (w/w) TritonX-100. Solution-G: A mixture of 3% (w/w) sodium alginate and 0.1% (w/w) TritonX-100. TritonX-100 was added to balance the surface tension between Solution-F and Solution-G.

Self-propulsion of the catalytic micromotors

The synthesized microparticles were put into a glass petri dish filled with 10 ml of 15% (w/w) H_2O_2 (Wako Pure Chemical Industries), 0.0005% (w/w) benzalkonium chloride (Wako Pure Chemical Industries), and 1% (v/v) isopropanol solution (Wako Pure Chemical Industries), and observed using

a digital microscope. The addition of isopropanol reduced the surface tension and decreased the bubble lifetime. The addition of benzalkonium chloride also reduced the surface tension.

Reference

[1] K. Maeda, H. Onoe, M. Takinoue, S. Takeuchi, *Adv. Mater.* **2012**, *24*, 1340.

[2] N. S. Cheng, *Powder Technol.* **2009**, *189*, 395.
